# Pathological and computed tomography findings of lymphoepithelioma-like gastric carcinoma with epithelioid granulomas: A case report

**DOI:** 10.3892/ol.2012.1070

**Published:** 2012-12-12

**Authors:** SHUGUANG LIU, LONG JIN, XIAOYAN XU, NI LIN, BIN LEI, HONG SHEN

**Affiliations:** 1 Department of Pathology, School of Basic Medical Sciences, Southern Medical University, Guangzhou;; 2 Department of Pathology, Fujian Provincial Hospital, Fuzhou;; 3 Department of Pathology, People’s Hospital of Zhengzhou Attached to Southern Medical University, Zhengzhou;; 4 Huiqiao Department, Nanfang Hospital, Southern Medical University, Guangzhou, P.R. China

**Keywords:** lymphoepithelioma-like gastric carcinoma, Epstein-Barr virus, granuloma

## Abstract

Lymphoepithelioma-like carcinoma is a distinct entity among Epstein-Barr virus (EBV)-associated gastric carcinomas, characterized by the presence of a lymphoid stroma with small nests of cancer cells uniformly distributed throughout. Epithelioid granulomas with multinucleated giant cells are extremely rare in the tumor tissue. The presence of epithelioid granulomas with prominent lymphocyte infiltration is associated with a favorable prognosis. This lesion tends to form a bulging mass in the submucosa. In the present study, we report a lymphoepithelioma-like carcinoma of the stomach with epithelioid granulomas which appeared as a bulging mass in CT scanning and correlate it with the pathology. Clinicians and radiologists should recognize the features of such tumors.

## Introduction

Lymphoepithelioma-like gastric carcinoma (LELC) is a rare type of gastric carcinoma with characteristic clinicopathological features (1–3). The majority of these tumors have been revealed to be associated with Epstein-Barr virus (EBV) infection (4). The development of epithelioid granulomas, named sarcoid-like reactions, is extremely rare in LELCs (5). The mass lesion may be misdiagnosed as a lymphoma, gastrointestinal stromal tumor (GIST) or carcinoid tumor. In the present study, the pathological results and CT findings of an EBV-associated LELC with epithelioid granulomas are described.

## Case report

**Figure 1.**
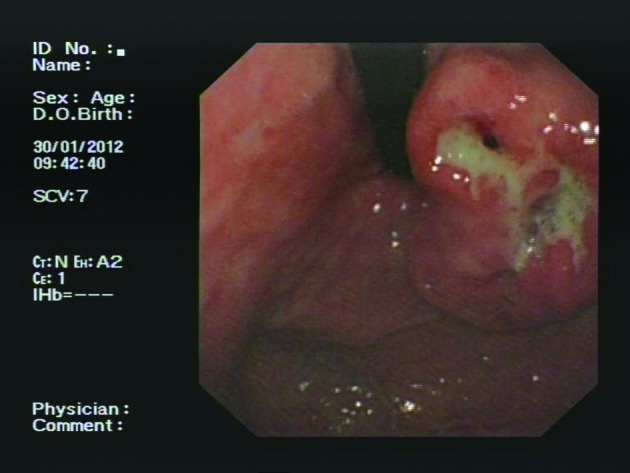
Endoscopic findings showing a submucosal mass in the lesser curvature of the upper body, with ulcers on the mucosal surface.

A 53-year-old Chinese male was admitted to Nanfang Hospital, Southern Medical University, Guangzhou, China, with a dull epigastric pain. Gastic endoscopy revealed a submucosal mass in the lesser curvature of the upper body with scattered ulcers on the mucosal surface (Fig. 1). The laboratory findings were unremarkable. A total gastrectomy was performed to remove the gastric mass. The patient remains alive with no evidence of recurrence after a five-month follow-up period.

**Figure 2.**
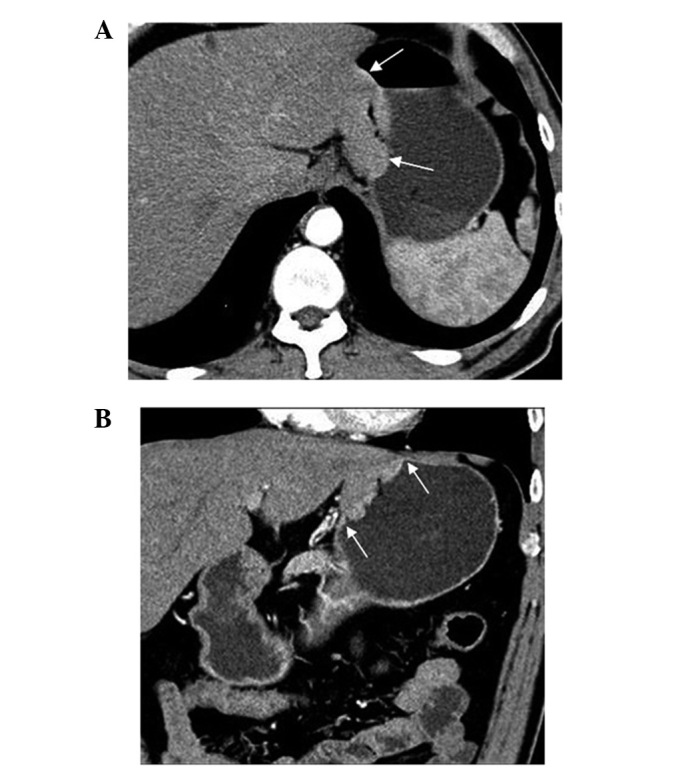
Contrast-enhanced CT scan and coronal reformatted images showed (A) a well-circumscribed mass in the lesser curvature with a large thickness-to-width ratio (arrows). (B) The low-density stripe of the normal gastric wall abruptly terminated at the edge of the lesion (arrows). The majority of the lesion was located in the submucosa according to pathological investigation.

A contrast-enhanced CT of the abdomen using a CT scanner (Siemens Somatom Plus 4; Siemens Inc., Munich, Germany) was performed at the hospital. The contrast-enhanced CT scan and coronal reformatted image showed a bulging mass at the lesser curvature wall of the gastric upper body near the cardia, with a large tumor thickness-to-width ratio (see arrows on Fig. 2A). The low-density stripe of the normal gastric wall abruptly terminated at the edge of the lesion (see arrows on Fig. 2B). There was no evidence of perigastric infiltration, enlarged lymph nodes or distant metastasis from the CT.

**Figure 3.**
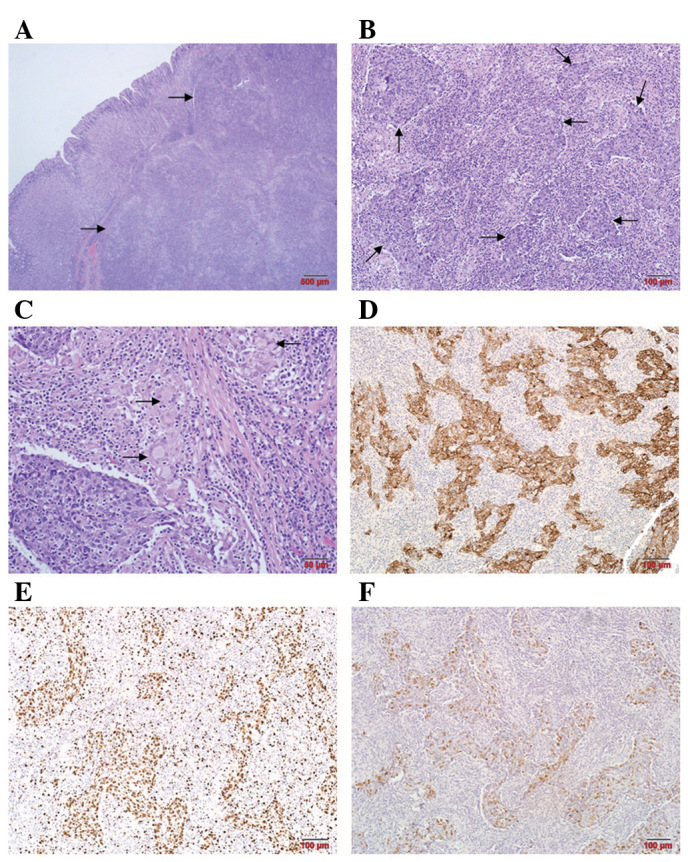
(A) The tumor was mainly located in the submucosa. Circumscribed mass was composed of lobulated nodules (arrows; hematoxylin and eosin stain). (B) Small nests of cancer cells were observed with no glandular structures (arrows). Marked infiltration of lymphocytes was observed in the tumor stroma (hematoxylin and eosin stain). (C) Epithelioid granulomas with Langhans type giant cells were noted in the lymphoid stroma (arrows; hematoxylin and eosin stain). (D) The tumor cells were strongly positive for Ckpan (immunohistochemical stain). (E) The expression of Ki67 in cancer cells was >90% (immunohistochemical stain). (F) Strong nuclear staining was observed in the nuclei of the cancer cells (EBER 1; *in situ* hybridization). EBER 1, Epstein-Barr virus encoded RNA 1.

Macroscopically, the resected specimen appeared as a 7.0×5.0×2.0-cm mass with ulcerations on the mucosa. Using light microscopy, a cross-section of the resected specimen at low magnification showed a circumscribed, superficially depressed ulcerated mass. The majority of the lesion was located in the submucosa (Fig. 3A). The tumor had invaded the subserosal adipose tissue. Histological examination revealed a poorly differentiated adenocarcinoma with marked peritumoral infiltration by lymphoid cells (Fig. 3B). Epithelioid granulomas with Langhans-type giant cells were noted in the lymphoid stroma (Fig. 3C). No lymph node metastasis was exhibited in 31 regional lymph nodes. Immunohistochemical staining indicated that the tumor cells were strongly positive for Ckpan (Fig. 3D). Immunohistochemistry for Ki67 revealed a high proliferation index (>90%; Fig. 3E). No expression of chromogranin (CgA), synaptophysin (SYN) or neuron-specific enolase (NSE) was observed (data not shown). *In situ* hybridisation for EBV-encoded RNA-1 (EBER-1) revealed strong nuclear staining in the tumor cells, while the background lymphocytes were negative (Fig. 3F).

## Discussion

EBV-associated gastric carcinoma is defined by the presence of EBV in gastric carcinoma cells and its absence in normal epithelium or dysplastic lesions (6). EBV-associated gastric carcinoma constitutes ∼8.7% of all cases of gastric carcinoma (3). It occurs in two histological patterns, LELC and ordinary gastric carcinoma. Of LELCs, 86–91% are positive for EBV, compared with ∼6% of diffuse and 7% of intestinal adenocarcinomas (3,6–9).

LELC, which constitutes ∼4% of all gastric carcinomas, is a relatively rare type of gastric carcinoma, characterized by morphological features similar to undifferentiated nasopharyngeal carcinoma (6). It occurs in old age, predominantly in males, and arises in the cardia or middle portion of the stomach. Pathological findings indicate that the cancer cells, which are arranged primarily in microalveolar, thin trabecular and primitive tubular patterns or as isolated cells, are surrounded by a dense population of non-neoplastic small lymphocytes (10–12).

LELCs are characteristically accompanied by prominent lymphocyte infiltration, particularly in the submucosa. EBV is considered to be the main cause of the lymphocytic response (13,14). The pathogenesis of the abundant lymphoid stroma in the submucosal layer remains unknown, but it is hypothesized that the formation of the submucosal mass is induced by the lymphocytic reaction. Granulomatous reactions, or sarcoid reactions, have been reported to occur in the regional lymph nodes of malignant tumors. However, numerous epithelioid granulomas with multinucleated giant cells are extremely rare in the tumor tissue, particularly of LELCs. To the best of our knowledge, only two studies have been published in English concerning epithelioid granulomas occurring in LELC of the stomach (5,6). In the present case, there was no lymph node or distant organ metastasis. This is because the spread of tumors through the gastric wall may be prevented by abundant lymphocytic reactions and granulomatous reactions. LELC has a more favorable prognosis than other forms of EBV-associated gastric carcinoma, as well as ordinary gastric carcinomas, although the tumor cells are often of the poorly differentiated type (14,15). The presence of epithelioid granulomas in the stroma with prominent lymphocyte infiltration may represent a host defense reaction against the cancer and is recognized as a favorable prognostic factor with regard to the immune response to the tumor (5).

Although LELCs have distinct clinicopathological features, they are not familiar to most radiologists. The CT appearance of LELCs with epithelioid granulomas has not been reported previously. In the present case, the advanced lesion was located in the gastric upper body near the cardia and appeared as a bulging mass with a large thickness-to-length ratio. The low-density stripe of the normal gastric wall abruptly terminated at the edge of the lesion. The majority of the lesion was revealed to be located in the submucosa by pathological investigation. It has been reported that the growth pattern with a larger thickness-to-length ratio is the characteristic appearance of EBV-associated lesions (16). Various factors, including epithelioid granulomas and lymphocytic induction, may contribute to the formation of the submucosal mass. Maeda *et al* reported that EBV-associated gastric carcinomas had various appearances in a CT study, including focal mucosal thickening, marked wall thickening with contrast enhancement and bulky portions projecting from the gastric wall (17). It is difficult to differentiate between LELCs presenting as a bulging mass and lymphomas, GISTs, neurogenic tumors and glomus tumors by imaging alone. It has been reported that the ulcerated shape of advanced EBV-associated gastric carcinomas is associated with the superficial depressed shape of the early lesions (18). However, in the present case, the tumor tissue with numerous epithelioid granulomas exhibited expansive growth and formed a nodule after invading the submucosa. The preoperative CT features may suggest the presence of LELCs. When LELC is suspected, detection of EBV may be performed using gastroscope biopsy specimens.

In summary, the CT findings of an LELC with epithelioid granulomas that developed in the gastric body revealed that it appeared as a bulging mass with abundant lymphoid stroma. The LELC tumor is a distinct entity associated with a good prognosis. The case in the present study is under follow-up at present. Therefore, understanding the radiological and clinical features of LELC is important in the preoperative diagnosis and in differentiating this entity from other tumors.
